# Bone and renal safety profile at 72 weeks after switching to tenofovir alafenamide in chronic hepatitis B patients

**DOI:** 10.1002/jgh3.12481

**Published:** 2020-12-19

**Authors:** Brian T Lee, Mimi Chang, Carolina Lim, Ho S Bae, Tse‐Ling Fong

**Affiliations:** ^1^ Asian Pacific Liver Center at Saint Vincent Medical Center Los Angeles California USA; ^2^ Division of Gastrointestinal and Liver Diseases Keck School of Medicine at University of Southern California Los Angeles California USA

**Keywords:** bone mineral density, chronic hepatitis B, proximal tubular function, switch, tenofovir alafenamide

## Abstract

**Background and Aim:**

Tenofovir disoproxil fumarate (TDF) has been efficacious in treating chronic hepatitis B (CHB), but long‐term use is accompanied by a decline in renal function and bone mineral density (BMD). Tenofovir alefanamide (TAF) is a prodrug of tenofovir, with similar efficacy in CHB but with fewer side effects than TDF. Recent studies on patients who underwent the switch from TDF to TAF have shown improved bone and renal profiles from 24 to 48 weeks of follow‐up.

**Methods:**

This study provides follow‐up at 72 weeks in a real‐world cohort of 61 Asian CHB patients who were switched from TDF to TAF. All patients had been treated with TDF for at least 12 months with hepatitis B virus DNA <21 IU/mL prior to switch.

**Results:**

Improvements in proximal tubular function, measured by urine beta‐2‐microglobulin to creatinine and retinol‐binding protein to creatinine ratios, were sustained at 72 weeks (*P* < 0.01). Renal function showed decline at 72 weeks compared to baseline (GFR_CG_ 90.9 *vs* 96.3 mL/min, *P* < 0.01). Improvement in hip BMD was sustained at 72 weeks (mean % change of 17.7% from baseline, *P* < 0.01). However, spine BMD showed discordance, with initial improvement at 24 weeks (3.3% from week 0, *P* < 0.01) but regression at 72 weeks (−0.6% from week 0, *P* = NS). Interestingly, there was a slight increase in weight and BMI after 72 weeks (*P* < 0.01).

**Conclusions:**

CHB patients who switch from long‐term TDF to TAF therapy show sustained improvement in proximal tubular function and hip BMD. Weight gain was noted, and long‐term studies are needed to evaluate its effect on patient outcomes.

## Graphical abstract

Long‐term follow‐up of CHB patients who switched from tenofovir disoproxil fumarate to tenofovir alefanamide continued to show efficacy in hepatitis B virus suppression along with an improved renal and bone safety profile. Improvement in proximal renal tubular function and bone density was seen as early as week 12 after the switch, and it appeared to have a “ceiling effect” at week 24. However, this improvement was sustained at week 72.

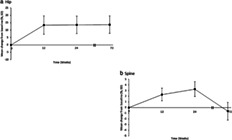

## Introduction

Chronic hepatitis B (CHB) viral infection remains a major global public health condition affecting more than 350 million individuals, with up to 40% developing significant clinical consequences, including cirrhosis, liver failure, or liver cancer.^1^, ^2^ Unable to achieve a “complete cure” of the hepatitis B virus (HBV) through the elimination of covalent closed circular DNA in hepatocytes, long‐term suppression using potent nucleos(*t*)ide analog antivirals remains the mainstay therapy in the treatment of CHB.^3^ Given the need for long‐term therapy, an antiviral agent with low risk of long‐term drug‐related toxicities is imperative for the overall health of patients with CHB. Tenofovir disoproxil fumarate (TDF) is a nucleotide analog with a high genetic barrier that is highly effective in achieving suppression with no reported resistance.^4^ However, long‐term use of TDF has been associated with the risk of renal dysfunction and reduction in bone mineral density (BMD).^5^

Tenofovir alafenamide fumarate (TAF) is a novel prodrug that reduces tenofovir plasma levels by 90% while having a similar efficacy as HBV suppression but reduced rates of BMD loss and renal toxicity when compared to TDF.^6^, ^7^, ^8^ However, these initial studies did not evaluate the outcomes in patients taking TDF and switching to TAF, a clinical situation that is commonly encountered, when considering long‐term therapy in patients with CHB. In a real‐world cohort of Asian patients with CHB, we previously showed an improvement in BMD and proximal tubular markers within 12 weeks of switching from TDF to TAF, which was sustained at week 24.^9^ Recently, several studies show similar improvement in bone and renal safety after switching from long‐term treatment with TDF to TAF with follow‐up of 24–48 weeks.^10^, ^11^ However, few studies have evaluated long‐term follow‐up of CHB patients after this switch to determine whether these improvements in renal and bone parameters are sustained. This current report presents the efficacy and the effects on BMD, renal glomerular, and renal tubular function of CHB patients who were treated with TDF for the long term and were switched to TAF (www.clinicaltrials.gov identifier NCT02957994) and followed for 72 weeks after switching.

## Methods

### *Study design and patients*

This is a prospective single‐arm open‐label study that started in March 2017 at the Asian Pacific Liver Center (APLC) at Saint Vincent Medical Center in Los Angeles, California. Each patient provided informed consent prior to enrollment. Adults (≥18 years) with CHB who were treated with TDF for at least 1 year with HBV DNA < 20 IU/mL for >6 months were invited to participate. Patients previously treated with adefovir or had a coinfection with human immunodeficiency virus (HIV) or hepatitis C virus were excluded. Patients with a history of organ transplantation were also excluded. Patients had an initial follow‐up of 24 weeks after the switch, with results reported in a previous study.^9^ These patients were subsequently followed up for 72 weeks after the switch.

Patients who met the inclusion criteria were switched from TDF to 25 mg of TAF orally once daily, administered with food. Postbaseline study visits occurred at weeks 12, 24, and 72. At each visit, a history and physical examination were obtained, while compliance was determined by pill count. Laboratory tests included a comprehensive metabolic panel, serum phosphorus level, uric acid level, and HBV DNA. Markers of tubular function obtained included urine albumin to creatinine ratio, fractional excretion of uric acid, fractional excretion of phosphate, and tubular maximum for phosphate corrected for glomerular filtration rate (GFR) (TmPO_4_/GFR). These tests were obtained at day 0, week 12, week 24, and week 72. Patients underwent BMD testing at screening, week 12, week 24, and week 72 of treatment. BMD in the anteroposterior view of the lumbar spine (from L1 to L4) and hip was measured using a Hologic QDR Discovery C enhanced‐array whole‐body DEXA scanner and version 12.1 software (Hologic Inc., Waltham, MA, USA). All scans were conducted on the same machine with a single operator. No major changes were made to other medications during the time of switch.

### *Outcomes*

Safety end‐points included changes in markers of renal function and percentage changes in hip and spine BMD at weeks 12, 24, and 72 compared to baseline. The efficacy end‐point included the proportion of patients with sustained HBV DNA less than 20 IU/mL.

### *Statistical analysis*

Baseline characteristics and laboratory values were described as means (standard deviation), medians (range), or frequencies (percentages). Laboratory values were compared by treatment status (before switch and at weeks 12, 24, and 72 of therapy). BMD values were also reported by mean percentage changes from baseline. Paired sample t‐test or Wilcoxon signed‐rank test was used to compare differences as appropriate. McNemar's test was used to compare proportions as appropriate. All statistical analyses were performed using R 3.6.1 (R Foundation for Statistical Computing, Vienna, Austria).

## Results

**Figure 1:**
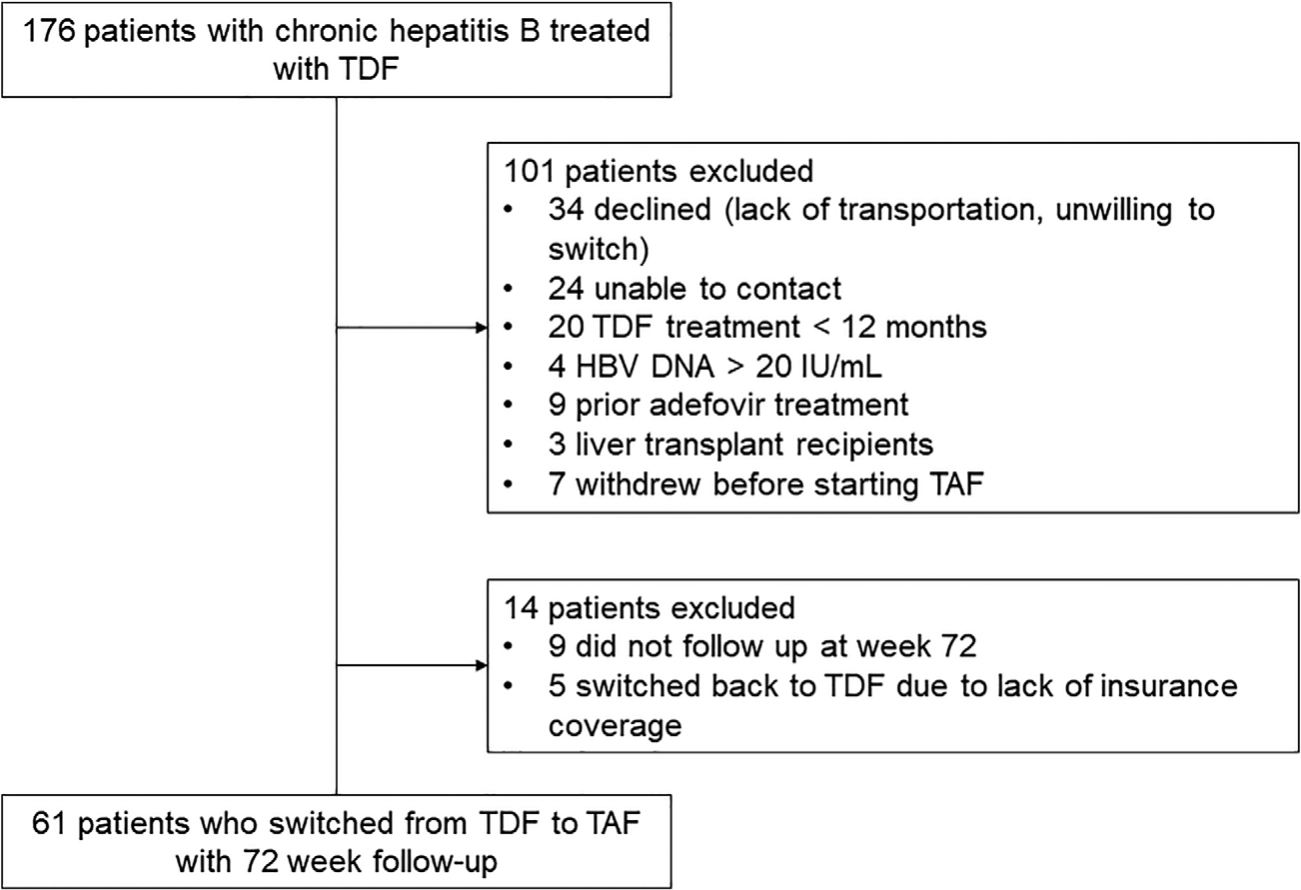
Flowchart of study cohort included in the analysis with follow‐up at 72 weeks. TAF, tenofovir alefanamide; TDF, tenofovir disoproxil fumarate.

From the previous cohort of 75 patients,^9^ 14 patients were excluded (9 were lost to follow‐up at 72 weeks, and 5 were excluded because patients returned to TDF due to insurance issues), resulting in a final cohort of 61 patients (Fig. 1). The median age was 57 (range 29‐83) years. Patients were mostly male (59%), and all were Asian (100%). The median duration of TDF therapy prior to switch was 56 (range 14–128) months. Of patients, 21% had hypertension, and 7% had diabetes mellitus. During the study, fasting blood glucose and blood pressure remained controlled with medical therapy. Of the 25 female patients in this study, 20 were postmenopausal. There were no patients with decompensated liver disease, and none decompensated during the study period. None were on diuretic therapy.

### *Renal safety*

At baseline, 36 patients (59%) had normal renal function as estimated by GFR_CG_ > 90 mL/min, while 19 patients (31%) had GFR_CG_ between 60 and 90 mL/min, and 6 patients (10%) had GFR_CG_ between 30 and 59 mL/min. When stratified by age, those 50 years or older had lower GFR_CG_ compared to younger patients (89.4 *vs* 111.5 mL/min). There were no differences in the prevalence of diabetes, hypertension, or time on TDF prior to switch between GFR_CG_ groups. Creatinine clearance by Cockcroft‐Gault was unchanged at week 24 but declined at week 72 (96.3 *vs* 94.4 *vs* 90.9 mL/min at weeks 0, 24, and 72, respectively; *P* < 0.01 for week 0 *vs* week 72, *P* < 0.05 for week 24 *vs* week 72) (Table 1). After 72 weeks, two patients with chronic kidney disease (CKD) stage 2 progressed to CKD stage 3, while one patient improved to stage 1. One patient with CKD stage 3 at screening improved to CKD stage 2 at the end of 72 weeks (Table 2).

**Table 1:** Demographics, renal parameters, and bone density measurements at weeks 0, 24, and 72

	Week 0	Week 24	Week 72
Age (years)	57 (29–83)		
Age group ≥ 50 years (%)	69		
Gender (% male)	59		
Race (% Asian)	100		
Weight (kg)	66.6 ± 12.1	67.7 ± 11.9**	68.1 ± 12.4**
Body mass index (kg/m^2^)	24.1 ± 3.4	24.5 ± 3.5**	24.7 ± 3.7**
Duration of TDF (months)	56 (14–128)		
Median ALT (U/L)	24 (9–88)	22 (9–64)	20 (10–156)
Prevalence of abnormal ALT^†^ (%)	26	23	20
Serum creatinine (mg/dL)	0.81 ± 0.18	0.82 ± 0.16	0.86 ± 0.17**^,^***
Creatinine clearance by Cockcroft‐Gault (mL/min)	96.3 ± 29.3	94.4 ± 27.3	90.9 ± 30.0**^,^***
Percent change from week 0 (%)		−0.6 ± 13.4	−5.2 ± 12.2**^,^****
Serum phosphorus (mg/dL)	3.2 ± 0.4	3.1 ± 0.4**	3.1 ± 0.5
Urine phosphorus (mg/dL)	51.4 ± 29.7	56.3 ± 33.1	54.5 ± 34.8
Fractional excretion of phosphate (%)	11.9 (4.5–41.7)	14.1 (6.0–30.4)*	13.4 (5.4–28.4)
Phosphate threshold for renal tubular reabsorption (mg/dL)	2.8 ± 0.5	2.6 ± 0.4**	2.7 ± 0.5
Abnormal (<2.8 mg/dL)	44	62*	57
Percent change from week 0 (%)		−5.1 ± 18.3*	−1.9 ± 30.0
Serum uric acid (mg/dL)	5.1 ± 1.0	5.4 ± 0.9	5.4 ± 0.9
Urine uric acid (mg/dL)	44.4 ± 23.5	49.7 ± 21.6	47.6 ± 22.8
Fractional excretion of uric acid	7.0 (0.6–29.0)	7.7 (1.6–17.0)	7.1 (0.6–14.4)
Urine albumin (mg/L)	3.8 (0.8–154.0)	4.6 (1.0–218.1)	5.2 (0.2–387.8)
Urine albumin/Cr ratio (mg/g)	0.04 (0.009–1.9)	0.04 (0.02–2.5)	0.05 (0.01–3.6)
Urine Beta‐2‐Microglobulin (μg/L)	155 (10–51 169)	102 (0–3991)**	120 (10–2578)**
Urine Beta‐2‐Microglobulin/Cr Ratio (μg/g)	1.5 (0.1–770.6)	1.2 (0–52.6)**	1.1 (0.07–27.9)**
Abnormal (>300 μg/g) (%)	2	0	0
Urine RBP (μg/L)	191 (39–43 600)	149 (18–1380)**	146 (18–1200)**
Urine RBP/Cr Ratio (μg/g)	1.7 (0.7–656.6)	1.5 (0.4–20.0)**	1.4 (0.1–13.0)**
Abnormal (>172 μg/g) (%)	2	0	0
Total Hip T‐Score	−1.2 ± 1.1	−0.8 ± 1.0**	−0.8 ± 1.1**
Total Hip BMD (g/cm^2^)	0.782 ± 0.191	0.860 ± 0.183**	0.891 ± 0.160**
Percent change from week 0 (%)		13.5 ± 23.2**	17.7 ± 23.2**
Lumbar Spine T‐Score	−1.4 ± 1.5	−1.2 ± 1.5**	−1.4 ± 1.6
Lumbar Spine BMD (g/cm^2^)	0.910 ± 0.170	0.937 ± 0.166**	0.902 ± 0.168****
Percent change from week 0 (%)		3.3 ± 5.2**	−0.6 ± 6.2****

^†^ Normal ALT cutoffs for male 35 U/L, female 25 U/L.

Mean ± standard deviation or median (range). Bolding indicates significant values. *n* = 61. *P < 0.05, **P < 0.01 compared to week 0, ***P < 0.05, ****P < 0.01 compared to week 24.

β2M, beta‐2‐microglobulin; ALT, alanine aminotransferase; BMD, bone mineral density; RBP, retinol‐binding protein; TDF, tenofovir disoproxil fumarate.

**Table 2:** Change in chronic kidney disease staging after 72 weeks

	Baseline		
	Stage 1	Stage 2	Stage 3
At Week 72			
Stage 1	27 (75%)	1 (5%)	0
Stage 2	9 (25%)	16 (84%)	1 (17%)
Stage 3	0	2 (11%)	5 (83%)

Chronic kidney disease stages were defined by GFR_CG_ (mL/min): Stage 1: ≥90; Stage 2: ≥60 and <90; Stage 3: <60.

There had been an improvement in markers of proximal tubular function by urine beta‐2‐microglobulin (β2M) (1.5 *vs* 1.2 μg/g, *P* < 0.01) and urine retinol‐binding protein (RBP) (1.7 *vs* 1.5 μg/g, *P* < 0.01) at 24 weeks compared to baseline.^9^ While these values did not change significantly from week 24 to week 72, the initial improvement was sustained at 72 weeks compared to baseline (*P* < 0.01) (Table 1). There was no change in urine albumin/creatinine ratio or fractional excretion of uric acid during the entire 72 weeks.

At week 24, serum phosphorus levels had decreased (3.1 *vs*.3.2 mg/dL, *P* < 0.01) with an associated increase in fractional excretion of phosphate compared to baseline (14.1 *vs* 11.9%, *P* < 0.05). The phosphate threshold for renal tubular reabsorption (TmPO_4_) also declined at week 24 compared to baseline (2.6 *vs* 2.8 mg/dL, *P* < 0.01). At week 72, all parameters of renal phosphate handling were reverted and were no longer significantly different from baseline (Table 1).

### *Bone mineral density*

**Figure 2:**
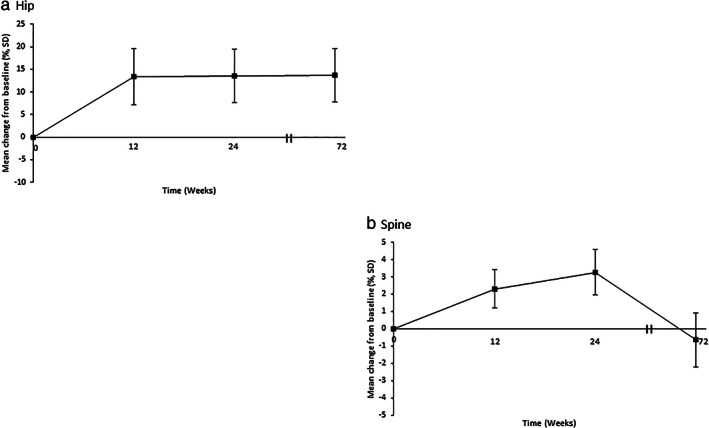
Mean percentage change from baseline to weeks 12, 24, and 72 in hip bone mineral density (a) and lumbar spine bone mineral density (b) by dual‐energy x‐ray absorptiometry. Error bars show 95% confidence intervals.

There was an improvement in BMD at week 24 compared to screening by measurements at both hip and lumbar spine sites.^9^ This improvement was sustained in hip measurements at 72 weeks (Fig. 2a). However, lumbar spine measurements declined at week 72 compared to week 24 and were no longer different compared to baseline (Table 1) (Fig. 2b).

### *Weight*

The mean body mass index (BMI) at screening was 24.1 ± 3.4 kg/m^2^. Based on both the World Health Organization and Asia‐Pacific guidelines, 20 patients (33%) were overweight, and 3 patients (5%) were obese. At week 24, there was an increase in BMI (24.5 ± 3.5 kg/m^2^, *P* < 0.01), which remained elevated at week 72 compared to BMI at screening (24.7 ± 3.7 kg/m^2^, *P* < 0.01) (Table 1). By the end of the study, 20 patients (33%) were overweight, and 5 patients (8%) were obese. There was no increase in incidence of hypertension or diabetes mellitus in this cohort after 72 weeks. Twelve patients (20%) gained 5% or more of their baseline weight at week 72.

### *Efficacy*

At baseline, 16 patients (26%) had abnormal serum alanine aminotransferase (ALT) levels. Of 16 of those with abnormal ALT, 6 were overweight/obese, while 1 patient was diabetic. There were no significant changes in ALT throughout the study. Of these 16 patients, 4 had normalization of serum ALT at 72 weeks. None of the patients with normal ALT level at baseline developed abnormal ALT level at 72 weeks. After 24 weeks of switch from TDF to TAF therapy, there were two patients with detectable HBV DNA levels. In both patients, virologic relapse was due to noncompliance that was corroborated by pill count.

## Discussion

Due to its antiviral potency and high genetic barrier, TDF is one of the recommended first‐line agents for treatment of CHB^3^. However, TDF is associated with renal toxicity and reduction in BMD. TAF is a novel prodrug that achieves intrahepatic levels of tenofovir efficiently with only 10% of plasma concentration of tenofovir compared to TDF, resulting in lower incidences of renal dysfunction and bone loss. With many CHB patients currently being treated with TDF, understanding the long‐term effects of switching from TDF to TAF is important to implement this into clinical practice. Most postmarketing studies on the TDF to TAF switch have primarily been in HIV patients, and few have evaluated long‐term outcomes in CHB patients.^10^, ^11^ This study extended one of the first “real‐world” studies in switching from TDF to TAF in clinical practice with a long‐term follow‐up of 72 weeks.^9^ The improvement in proximal renal tubular function and BMD shown at week 12 was sustained through week 72, confirming the improved safety profile of TAF in patients previously treated long‐term with TDF. In addition, the efficacy of HBV suppression after switching from TDF to TAF remained excellent at 72 weeks of follow‐up, with an overall rate of 97%.

Compared to baseline, proximal renal tubular dysfunction, including urine β2M to creatinine ratios and RBP to creatinine ratios, improved significantly at week 24 from the initial switch. However, there was no further improvement between weeks 24 and 72, although the improvement from baseline was sustained. Our study suggests that the improvement in renal tubular parameters following the switch occurred during the first few months, and there may be a “ceiling effect” in recovery after the initial toxicity of TDF. In a study by Agarwal and colleagues comparing TDF *versus* TAF with a follow‐up of 92 weeks, CHB patients receiving TDF compared to TAF had greater mean percentage changes at week 96 for urine RBP to creatinine ratio (103.4 *vs* 46.7%, respectively, *P* < 0.001) and urine β2M to creatinine ratio (297 *vs* 66.5%, respectively, *P* < 0.001).^6^ Similarly, Lampertico and colleagues evaluated CHB patients who were treated with at least 48 weeks of TDF and were randomized to switching from TDF to TAF or remaining on TDF with a follow‐up of 48 weeks. At week 48, patients who switched had improved median percentage change from baseline in urine RBP to creatinine ratio (−17.7%) and urine β2M to creatinine ratio (−36.0%). Interestingly, this study had an increase in grade 1 proteinuria after 48 weeks from switch (7 to 14%).^11^ Despite an attenuated response from weeks 24 to 72, our cohort showed an improvement in renal tubular changes compared to baseline in CHB patients receiving TAF.

Renal tubular absorption of phosphate (TMPO_4_/GFR) is another parameter used to assess proximal tubular function.^12^ To our surprise, our previous study showed a slight but statistically significant decrease in TmPO_4_ at week 24 after switching despite improvement in other markers of proximal renal tubular function. These changes were concurrent with a decrease in serum phosphorus levels and increase in fractional excretion of phosphate. However, after 72 weeks, this effect was no longer present, and renal parameters of phosphate handling and serum phosphate levels were no longer significantly different from baseline. At the end of follow‐up, there were no episodes of Fanconi syndrome in the study cohort.

While there was no difference in GFR_CG_ from screening to week 24, there was a notable decrease by the end of follow‐up at 72 weeks. In the previously mentioned study of CHB patients who continued TDF compared to switching to TAF, patients who stayed on TDF continued to show a decline in their GFR_CG_, while patients who switched to TAF had mild improvement. Similar to our patients, after switching from TDF to TAF, some patients continued to show decline in GFR_CG_ at the end of 48 weeks. Furthermore, after 24 weeks, there was a pattern of decline of renal function in both TDF and TAF arms. Two patients in the TAF arm had progression of CKD from stage 2 at baseline, with one progressing to stage 3 and the other to stage 4.^11^ In our cohort, 11% of patients with baseline CKD stage 2 progressed to stage 3. As the majority of our patients had relatively preserved renal function at screening, the effect of switching to TAF in those with poor renal function, such as those with CKD stage 4 or end‐stage renal disease, at baseline could not be characterized.

Our study showed that switching from TDF to TAF did not result in worsening BMD at long‐term follow‐up with sustained improvement in hip BMD at 72 weeks since switching therapy. While previously thought to be related to urinary phosphate loss, bone changes have been theorized to be related to tenofovir's effect on osteoblast and osteoclast activity.^13^, ^14^ In addition, a study on TDF to TAF switching in HIV patients showed a sharp decline in parathyroid hormone levels.^15^ A previous study showed changes in bone biomarkers (such as C‐terminal cross‐linking telopeptide for type 1 collagen, procollagen type 1 N‐terminal pro‐peptide, bone‐specific alkaline phosphatase) for both resorption and formation when comparing TDF and TAF. Patients with CHB receiving TAF had minimal changes in bone biomarkers compared to those receiving TDF, especially in bone resorption, where those receiving TDF appeared to have a catabolic window for bone turnover.^14^ In our study, while there was improvement seen in lumbar spine BMD at 24 weeks, this effect was no longer present at 72 weeks. The effect of TDF on BMD loss has been especially seen in measurements of the hip compared to lumbar spine.^6^, ^14^ Discordance of hip and lumbar spine measurements by DEXA can be seen, especially in increasing age and after menopause.^16^ Of the 25 female patients in this study, 20 were postmenopausal.

Interestingly, our cohort of CHB patients showed a minor but significant weight gain during the 72‐week period after switching from TDF to TAF. Recent studies in switching from a TDF to TAF regimen in HIV patients have shown more appreciable increases in weight gain in long‐term follow‐up.^17^, ^18^, ^19^ The reason for this weight gain while taking TAF was unclear, but it was hypothesized to be related to an interplay of HIV‐specific factors and antiretroviral therapy such as integrase inhibitors, which is not applicable in our study. We did not evaluate of the effect of weight gain on metabolic factors or development of hepatic steatosis.

The strength of this study is that it reflects a “real‐world” experience of Asian‐American patients with CHB and compensated liver disease who were treated with TDF for a long time prior to switching to TAF. Our study is limited by being a single‐arm study with solely Asian patients. Weight patterns during treatment duration of TDF were not measured. In addition, long‐term TAF use and its effect on renal function in decompensated cirrhosis (i.e. ascites including the use of concomitant diuretics, variceal hemorrhage, jaundice) was not assessed in this study. Thus, our results cannot be extrapolated to this patient population.

Long‐term follow‐up of CHB patients who switched from TDF to TAF continued to show efficacy in HBV suppression along with an improved renal and bone safety profile. Improvement in proximal renal tubular function and bone density was seen as early as week 12 after switch, and it appeared to have a “ceiling effect” at week 24. However, this improvement was sustained at week 72. Our study of a cohort of real‐world patients with CHB shows the long‐term benefit in switching from TDF to TAF. Future studies should evaluate the aspect of weight gain and its clinical implications after switching from TDF to TAF.
